# Reflective mindfulness and emotional regulation training to enhance nursing students’ self-awareness, understanding, and regulation: a mixed method randomized controlled trial

**DOI:** 10.1186/s12912-025-03086-w

**Published:** 2025-04-30

**Authors:** Gihan Mohamed Mohamed Salem, Wilf Hashimi, Ayman Mohamed El-Ashry

**Affiliations:** 1 Psychiatric and Mental Health Nursing Department, Faculty of Nursing, Benha University, Banha, Egypt; 2 Metanoia Institute, Middlesex University, 13 North Common Road, Ealing, London, W5 2QB UK; 3 Psychiatric Nursing and Mental Health, Faculty of Nursing, Alexandria University, Alexandria, Egypt

**Keywords:** Mindfulness, Emotional regulation, Nursing students, Nursing education, Self-awareness, Mixed method, Randomized controlled trial

## Abstract

**Background:**

Nursing students encounter significant academic and psychological challenges that can impede their transition from theoretical knowledge to practical application, affecting their well-being and professional development.

**Objective:**

This study aimed to evaluate the effectiveness of Reflective Mindfulness and Emotional Regulation Training (RMERT) in improving nursing students’ self-awareness, understanding, and emotional regulation.

**Design and methods:**

This study employed a convergent parallel mixed-method randomized controlled trial design. Forty fourth-year undergraduate nursing students were randomly assigned using simple randomization (computer-generated random numbers) into either an intervention group (*n* = 20) or a control group (*n* = 20). The intervention group participated in a six-week RMERT program designed to enhance self-awareness, understanding, and emotional regulation. The control group continued with standard course activities. Quantitative data were collected pre- and post-intervention using the Emotion Regulation Questionnaire (ERQ) and the Mindful Attention Awareness Scale (MAAS), and analyzed using SPSS. Qualitative data, gathered exclusively from the intervention group through recorded reflective group sessions, were analyzed using thematic analysis.

**Results:**

The intervention group exhibited significantly improved self-awareness, understanding, and emotional regulation compared to the control group. Additionally, students displayed an increased capacity for mindfulness, a decreased tendency to suppress emotions, and greater comfort in reflecting on positive and negative emotions.

**Conclusion:**

Integrating RMERT into nursing curricula may enhance nursing students’ well-being and professional growth, mainly when counseling resources are limited. The program can improve self-awareness, understanding, and regulation skills necessary for effective nursing practice.

**Implication for nursing practice:**

Integrating RMERT into nursing curricula enhances self-awareness, emotional regulation, and reflective practice. This approach equips students with essential skills for managing clinical stress and building therapeutic relationships. As future nurses, they develop resilience, clinical reasoning, and empathetic patient care, ultimately elevating care quality and professional growth in diverse healthcare settings.

**Trial registration number:**

The study was registered retrospectively on https://register.clinicaltrials.gov/ on 23 of December 2024 under the reference number: NCT06760962.

## Introduction

Undergraduate nursing students must acquire advanced professional knowledge and clinical competencies to establish therapeutic relationships with diverse clients and provide high-quality care [1, 2]. Such competencies are critical for both personal and professional development since they equip nursing students to manage the rigors of their coursework and clinical training and prepare them for future healthcare careers [1, 3]. Nursing students reported in various studies [4, 5] that mental health nursing is one of the most anxiety-inducing and stressful courses, making them more likely to encounter challenging circumstances and experience high levels of stress. Students described such stress/ anxiety as having negative impacts on their mental, psychological, and often physical well-being; consequently, their academic and practical performance, attendance in classes, and interpersonal relationships are adversely affected [6]. One of the most challenging skills described for mastery was cultivating a mindful mindset, which encompasses self-awareness and understanding as well as comprehending and regulating emotions [7].

According to [7, 8], these skills are essential for establishing and maintaining therapeutic interactions with patients, exhibiting empathy, and engaging oneself as a therapeutic tool. Students with undiagnosed or untreated psychological or mental health issues may find these skills incredibly challenging and potentially overwhelming. Since some students may already be experiencing or developing severe problems, failing to address these and students’ probable vulnerability in this clinical field puts them at considerable risk [6].

Additionally, the stigma and shame of psychological/ mental health issues often mean that students feel ashamed and reluctant to admit to them or to seek support from tutors or counseling/psychotherapy professionals [9]. Moreover, there are still societies like the one where the study was conducted, lacking a clear understanding of the actual role of counseling/ psychotherapeutic services in addressing psychological/ mental issues [9, 10].

### Mindfulness and emotional regulation in nursing education

Although mindfulness is natural, practice is needed to master it for functional use [6, 7]. For nursing students to be competent in their academic and clinical experiences, they need to develop self-awareness, self-understanding, and self-regulation skills [11, 12]. These skills help nursing students adopt the highest moral and professional standards while fulfilling the demands of their coursework’s academic and clinical training [11].

A mindful mindset, accessing emotional/mental states, and learning to accept, process, and regulate them are essential skills and strategies for nursing students to develop [12]. The techniques people use to regulate their emotional responses or those of others are called “emotional regulation” [13]. Two common strategies are “cognitive reappraisal,” which is considered healthy, and “expressive suppression,” which is regarded as maladaptive: cognitive reappraisal leads people to change the way they think about emotionally related events, whereas suppression means the inhibition of emotions [13, 14].

### Reflective psychoeducational program

Reflection is an insightful teaching and learning approach in nursing education that fosters students’ growth on both personal and professional levels. It helps students get a better sense of self-awareness and understanding by recognizing their areas of strength, development, and weakness, as well as their knowledge and skill gaps [15, 16]. Critical evaluation of their experiences, including thoughts, feelings, behaviors, personal values, and beliefs, is another way it forces students to grow and overcome challenges in their clinical practice [17].

Reflective psychoeducation programs promote self-reflection, insight-building, and critical appraisal of mental and emotional states and restrictions [17, 18]. Such programs contribute to cognitive reappraisal by providing space and process for undergraduate nursing students (hereafter “the students”) to become more mindful as they reflect on and regulate their mental/emotional processes while experiencing the support that comes with being in a therapeutic relationship [18, 19]. Schön [20] highlights the crucial role of reflection in professional practice and distinguishes between two types of reflection: “reflection-on-action” and “reflection-in-action.” Reflection-on-action is reviewing and analyzing past experiences to gain insight and improve future decisions and actions [21]. On the other hand, Reflection-in-action is the process of reflecting while practicing that enables the individual to think critically, adapt in challenging and unpredictable circumstances, and make immediate choices and adjustments [20, 22].

Furthermore, Edwards (2017) discussed how the potential advantages of reflection are limited in nursing education when the emphasis is mostly on reflection following acts or reflection-on-action. Nurses may achieve a more holistic approach to professional practice and improved learning from practice through the adoption of a four-dimensional reflection process: before, during, after, and beyond actions [21].

The current study’s RMERT program is mainly based on cognitive behavioral therapy (CBT), and it adapts the empathic therapeutic techniques drawn from transactional analysis (TA) psychotherapy. CBT focuses on how cognition influences the expression of emotions and behaviors. It assumes maladaptive feelings and behaviors develop through maladaptive cognitive processes [23]. Thus, identifying these and learning new ways of perceiving and thinking about events will lead to more positive emotional and behavioral patterns [24]. Empathic transactions are TA therapeutic techniques that enable the structuring and analyzing what occurs throughout the therapeutic relationship [25].

The program mainly applied the three dimensions of reflection: before, after, and beyond, to train and equip students to apply the reflection in action later while in the clinical setting, where students were facilitated to express their struggle with recognizing, processing, regulating or verbalizing their mental and emotional states [15, 18].

This study innovatively integrates Transactional Analysis (TA) with Cognitive Behavioral Therapy (CBT) to address cultural-specific mental health challenges among Egyptian nursing students. While earlier research has primarily employed CBT for emotional regulation, our approach uniquely incorporates TA’s empathic techniques to structure therapeutic interactions and enhance self-reflection. This dual-method intervention fosters adaptive cognitive reappraisal while addressing culturally ingrained stigmas, providing a comprehensive framework for understanding and managing emotions. By combining these methodologies, our research offers a novel, culturally sensitive model that effectively refines existing therapeutic techniques and enriches students’ self-awareness and regulation skills [9, 10, 20].

In Egypt, deeply ingrained cultural beliefs play a significant role in shaping perceptions of mental health, often leading to stigma and misconceptions that hinder open dialogue and help-seeking behaviors [9]. Traditional views may attribute mental health challenges to supernatural causes or personal weakness rather than recognizing them as medical issues [10]. This cultural backdrop, combined with limited access to mental health resources and counseling services, exacerbates the emotional burdens faced by nursing students. These students often encounter significant academic and clinical stress while navigating societal norms that discourage the expression of vulnerability. RMERT becomes uniquely essential in this setting, as it equips future nurses with the skills to manage their emotions, challenge cultural stigmas, and develop resilience in high-pressure environments. Integrating RMERT into nursing curricula can foster enhanced self-awareness and emotional regulation, ultimately leading to improved patient care and professional growth [9, 20].

Thus, the current study broadens the body of knowledge of research supporting the incorporation of reflective psychoeducational programs into nursing education. Given the lack of randomized controlled trials and mixed methods research in the study’s setting, this study aims to evaluate the effectiveness of a reflective psychoeducational program grounded in mindfulness and emotional regulation strategies in enhancing self-awareness, self-understanding, and self-regulation in nursing students.

## Methods

### Study design

**Fig. 1:**
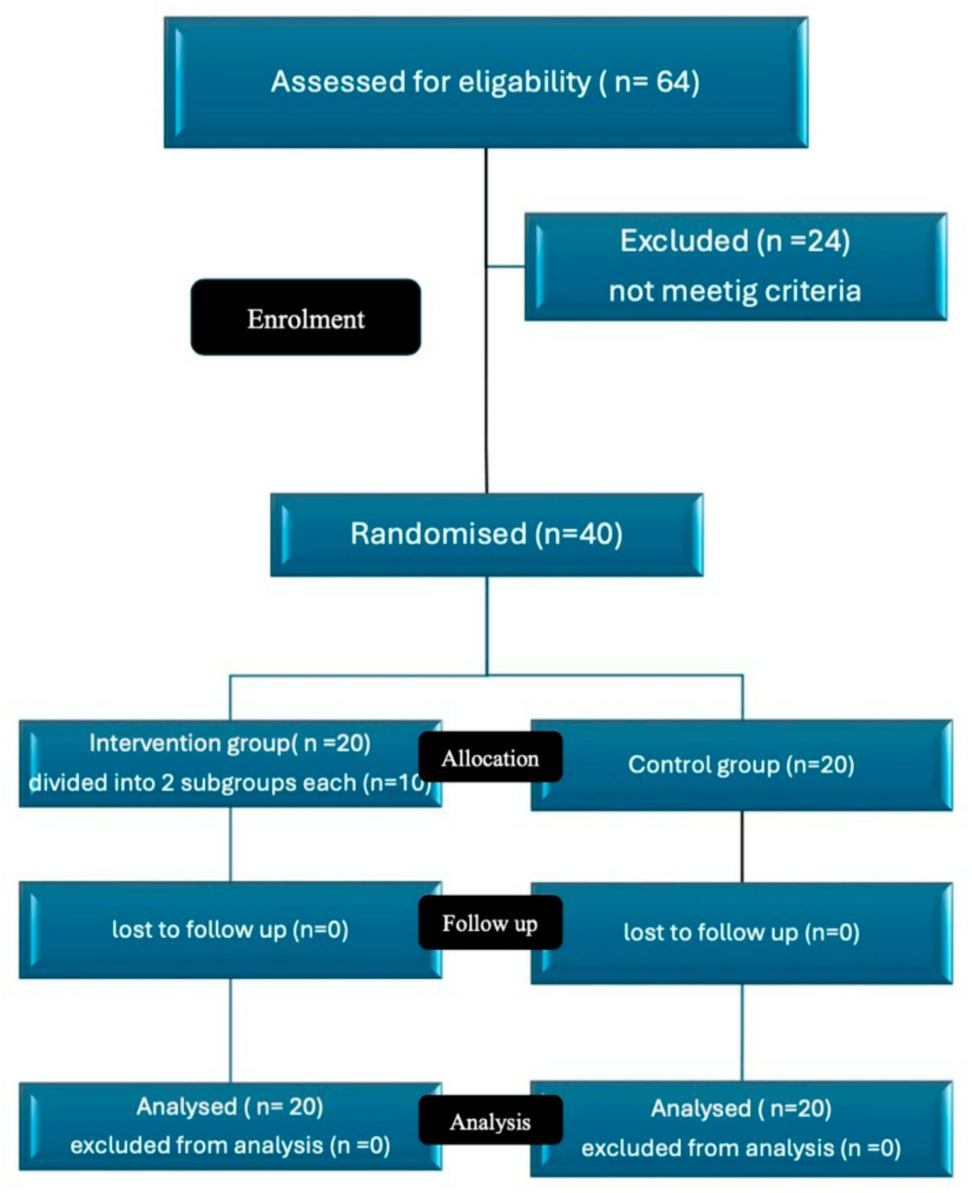
The CONSORT-SPI 2018 flow diagram

Study Design The current study employed a convergent parallel mixed-method design, integrating qualitative and quantitative methods simultaneously and independently to allow comprehensive insights into the phenomena under investigation. This two-arm parallel randomized controlled trial (RCT) was registered retrospectively on https://register.clinicaltrials.gov/ on 23 December 2024 (reference number: NCT06760962). The study followed the reporting guidelines for randomized trials of social and psychological interventions: CONSORT-SPI 2018 Extension (Fig. 1) [26]. The study followed mixed research methods and the quantitative results were analyzed with SPSS, while the qualitative results were submitted to thematic analysis [27].

### Sample size calculation

Students in the psychiatric and mental health nursing course in the faculty of nursing at Benha University were invited to participate in this study. In a post-hoc power analysis conducted for a study with predetermined study and control groups, each consisting of 20 subjects, the results indicated a power probability of 0.337. This analysis was based on degrees of freedom (df) of 38, with a critical t-value of 2.02 (see Fig. 1). The non-centrality parameter was calculated to be 1.58. These results suggest that the study, with the given sample size and effect size, has a relatively low power to detect a significant effect, indicating a higher likelihood of Type II error. However, we complement our quantitative findings with qualitative data to provide a richer context and deeper understanding of the phenomena under study. The data collection and the quantitative data analysis were conducted between 2023 and 2024.

Inclusion criteria: students enrolled in the psychiatric/mental health nursing course for the first time, i.e., did not enroll in or join similar or the same course before; for example, (a) joined the faculty from the secondary school, not through other paths or bridging educational programs that would include psychiatric/mental health courses/modules as part of their programs, (b) did not fail the course before or attended it for more time(s).

Exclusion criteria: (a) undertaking another program of self-health promotion; (b) undergoing mental health counseling; (c) undergoing psychopharmacological treatment during the study; or (d) displaying clear signs of significant emotional instability.

Participant Recruitment Fourth-year undergraduate nursing students enrolled in the psychiatric and mental health nursing course at Benha University were invited to participate in the study via email invitations and bulletin board announcements at the faculty premises. Clear instructions regarding the study’s aim, inclusion criteria, and participation expectations were provided. Interested students voluntarily contacted the research team to express their interest and receive further details.

### Random allocation

Forty students met the eligibility requirements and were randomly assigned at a 1:1 ratio to control and intervention groups of twenty each. The intervention set was then split into ten subgroups to form counseling groups. The intervention and control groups completed the pre-test and post-tests one week before the sessions began and one week after they ended. Students’ identifiers were removed to ensure confidentiality. In two subgroups of 10, each intervention group attended intervention sessions, while the control group did not receive any intervention sessions. During the intervention sessions, both groups attended the theoretical and clinical parts of the psychiatric mental health nursing course with their colleagues. As part of their training, they facilitated nursing services for actual patients in clinical settings.

The researchers ensured the control group did not receive any other potentially contaminating intervention or attend any promoting or supporting sessions, workshops, lectures, meetings, etc., during the study. Complete blinding was tricky in this study on Reflective Mindfulness and Emotional Regulation Training (RMERT) because the participants knew whether they were in the intervention or control group. The intervention group attended specific training sessions, while the control group did not, making it hard to hide who was receiving the training. However, to reduce bias, the researchers who analyzed the data needed to know which group the participants were in. Although we could not fully blind everyone, this helped ensure that the results were as fair and accurate as possible.

### Ethical considerations

Ethical approval was given by the faculty’s Research Ethics Committee with No: REC PSYN P39. Students were informed of their right to withdraw from the study at any time without consequences and that being absent for more than one session or not answering questionnaires thoroughly would result in exclusion from the study. After obtaining informed written consent, all students answered the questionnaires and the intervention groups attended all sessions. With the students’ agreement, sessions were recorded for subsequent thematic analysis. For ethical reasons, the rest of the students received the same intervention at the end of the study.

### Intervention group

The intervention consisted of six 90-minute weekly sessions. The RMERT program was developed and processed by two researchers: both are Certified Transactional Analysts in the field of psychotherapy and have CBT practitioner certificates, besides other certificates in counseling and psychotherapy. Additionally, the first practitioner/researcher who facilitated the program has a doctorate in Psychiatric Mental Health Nursing. The RMERT program was mainly based on CBT and supplemented with therapeutic techniques drawn from TA. The CBT aspect concentrated on the ABC model [23], where A represents Activating events, B stands for Beliefs held, and C indicates emotional and behavioral Consequences. The TA therapeutic techniques involved empathic transactions, including inquiry, specification, confrontation, confirmation, illustration, explanation, interpretation, holding, and crystallization [25]. Mindfulness relaxation techniques were also taught, such as muscle relaxation and diaphragmatic breathing [28]. The RMERT program is presented in Table 1.

**Table 1:** The RMERT program was presented as follows

Session 1	Concepts and techniques of mindfulness, emotional regulation, and dysregulation
Session 2	ABC model, including examples
Session 3	Reflection on therapeutic relationships using ABC model and emotional regulation techniques
Session 4	Reflection on mindfulness and relaxation practice experiences
Session 5	Applying ABC model to the theme of ending
Session 6	Summarizing and ending the program

## Measures

### Emotion regulation questionnaire

The ERQ is a 10-item self-report scale designed to assess two emotion regulation strategies: cognitive reappraisal (ERQ-R) (a healthy strategy) and expressive suppression (ERQ-S) (a maladaptive strategy) [14]. (a) Cognitive reappraisal of an emotional experience attempts to change how one thinks about a potentially emotion-inducing situation (6 items). (b) Expressive Suppression attempts to reduce or inhibit an emotional response to a situation (4 items). Respondents answer each item on a 7-point Likert-type scale ranging from 1 (strongly disagree) to 7 (strongly agree). The scoring takes the average of all the scores in each subscale. The higher the score, the greater the use of that strategy and vice-versa. The ERQ has a test-retest reliability of 0.69 for both sub-scales; the ERQ sub-scales have shown to be internally consistent among college-age students with reappraisal (α = 0.79) and suppression (α = 0.73).

### Mindful Attention Awareness Scale (MAAS)

The MAAS (Brown & Ryan, 2003) is a 15-item self-report scale designed to assess a core characteristic of mindfulness, namely, a receptive state of mind in which a sensitive awareness informs attention of what is occurring in the present [29]. Response options ranged from 1 (almost always) to 6 (rarely). The scale shows strong psychometric properties and has been validated with college, community, and cancer patient samples. The score is a simple mean of the 15 items, with higher scores indicating more highly developed mindfulness. The MAAS has high test-retest reliability. College-aged students’ internal consistency levels (i.e., Cronbach’s alpha) have been reported from 0.80 to 0.90.

In addition, socio-demographic data, including age, sex, residence (urban/rural), marital status, employment status, living arrangements (living with family or independently), and cumulative GPA, were collected from participants to provide a comprehensive participant profile.

### Data analysis

The collected data were categorized as quantitative and qualitative, as discussed below.

### Quantitative data

The quantitative data were tabulated and analyzed using SPSS software (version 16). Tests for normality using the Shapiro-Wilks test assuming normality at *P* > 0.05, as proved to be normally distributed, were expressed as mean ± standard deviation and analyzed by Student “t” test for two independent groups and the Paired “t” test for two matched variables pre- and post-intervention among the same group. The chi-square (X2) and Fisher’s exact tests were used to compare socio-demographic data among the studied groups. *P* ≤ 0.05 was considered statistically significant.

### Qualitative data

The qualitative data were collected through semi-structured individual interviews conducted primarily in a controlled, private setting at a designated research facility associated with a mental health center, with secure virtual platforms available when in-person sessions were not feasible. Each session, lasting approximately 60 to 90 min, was audio-recorded with participants’ informed consent, and detailed written field notes were maintained to capture non-verbal cues and contextual details; in some instances, video recordings were also employed to further enrich the dataset. The recorded sessions then underwent a comprehensive, iterative thematic analysis conducted closely with supervisors in psychiatric mental health nursing, research, and psychotherapy.

Thematic analysis was selected for its flexibility and systematic approach to identifying, analyzing, and reporting patterns within qualitative data [27]. Given the study’s focus on understanding nursing students’ experiences with reflective mindfulness and emotional regulation, thematic analysis allowed us to capture the richness and complexity of these narratives while identifying common themes that reflect shared perceptions and cultural influences. Unlike approaches such as grounded theory, which are geared towards developing new theoretical models, or phenomenological methods that focus deeply on individual lived experiences, thematic analysis efficiently provided a structured yet adaptable framework to explore and interpret the data in relation to our specific research questions. This method was particularly well-suited to our mixed-method design, as it facilitated the integration of qualitative insights with quantitative findings to enrich our understanding of the intervention’s impact.

This analysis followed six meticulously executed phases: Phase 1 involved familiarization with the data through transcription, repeated readings, and the generation of early ideas; Phase 2 focused on creating initial codes; Phase 3 entailed organizing these codes into potential themes; Phase 4 consisted of a thorough review where themes were compared to codes and mapped; Phase 5 was dedicated to defining and naming the themes, and Phase 6 culminated in the creation of a final report featuring vivid data extracts. Iterative discussions throughout the process ensured unanimous agreement on the final themes, and data saturation was confirmed when successive interviews ceased to reveal new themes—a conclusion further validated by the convergence of these qualitative insights with the findings from accompanying questionnaires.

## Results

### Quantitative data

#### Matching of socio-demographic and clinical data characteristics

Table 2 presents a detailed comparison of the demographic data between the study group (*n* = 20) and the control group (*n* = 20). The mean age for the study group was 20.83 ± 1.49, compared to 20.11 ± 1.15 for the control group, with a p-value of 1.000, indicating no significant difference. Regarding sex, 45% of the study group were male and 55% female, while the control group had an equal distribution of 50% male and 50% female participants, with a p-value of 0.787. Both groups had an identical distribution in residence, with 85% living in urban areas and 15% in rural areas, yielding a p-value of 1.000. Marital status was also consistent across the groups, with 95% being single and 5% married, with a p-value of 1.000. Employment status showed that in the study group, 60% were not working, 25% had nursing jobs, and 15% had non-nursing jobs, while in the control group, 65% were not working, 25% had nursing jobs, and 10% had non-nursing jobs, with a p-value of 0.859. Regarding living arrangements, 60% of the study group and 55% of the control group lived with family, with a p-value of 0.694. Finally, cumulative GPA distributions were identical, with a p-value of 0.437. This comprehensive comparison provides a clear understanding of the baseline characteristics of the study, enhancing the context for the subsequent results.

**Table 2:** Comparison between the two studied groups according to demographic data

	Study (*n* = 20)		Control (*n* = 20)		Test of Sig.	*p*
	*n*.	%	*n*.	%		
**Age**						
< 20	7	35	8	40	χ^2^ = 0.635	0.426
≥ 20	13	65	12	60		
Mean ± SD	20.83 ± 1.49		20.11 ± 1.15		t = 0.000	1.000
**Sex**						
Male	9	45	10	50	χ^2^ = 0.073	0.787
Female	11	55	10	50		
**Residence**						
Urban	17	85	17	85	0.000	^FE^p= 1.000
Rural	3	15	3	15		
**Marital Status**						
Single	19	95	19	95	χ^2^ = 0.000	^FE^p= 1.000
Married	1	5	1	5		
**Have job**						
Not work	12	60	13	65	χ^2^ = 0.407	^MC^p= 0.859
Nursing work	5	25	5	25		
Non nursing work	3	15	2	10		
**Home Participants**						
Alone	3	15	3	15	χ^2^ = 1.867	^MC^p= 0.694
With family	12	60	11	55		
With colleagues	5	25	6	30		
**Cumulative GPA**						
0 < 2	4	20	5	20	χ^2^ = 1.654	0.437
2 < 3	12	60	11	60		
3 < 4	4	20	4	20		

SD: Standard deviation t: Student t-test χ^2^: Chi square test MC: Monte Carlo FE: Fisher Exact

p: p value for comparing between the studied groups

#### Regarding students’ mindfulness

In Table 3, the results regarding students’ mindfulness are particularly intriguing. There was no statistically significant difference (*P* > 0.05) between the intervention and control groups regarding the mean values of pre-intervention average MAAS (1.75 and 1.76, respectively). However, a statistically significant difference (*P* < 0.001) emerged between both groups on post-intervention MAAS. Here, the mean value was statistically significantly higher for the intervention group (5.34) compared to the control group (1.53). This significant change in the mean values within each group, with the intervention group increasing from 1.75 to 5.34 and the control group decreasing from 1.76 to 1.53 (*P* ≤ 0.001), suggests a noteworthy impact of the intervention on students’ mindfulness. The decrease in mindfulness for the control group may be attributed to the inherent stressors and demands of their clinical and academic environment. Without the structured reflective practices and mindfulness training provided by RMERT, these students likely faced escalating stress levels that eroded their natural capacity for self-awareness and adaptive emotional reframing.

**Table 3:** Comparing the studied groups regarding MAAS pre- and post-intervention

Average MAAS	Intervention group (*N* = 20)			Control group (*N* = 20)			St.“t” test	*P* (significance)
	Mean	SD	Range	Mean	SD	Range		
Pre	1.75	0.17	1.47–2.13	1.76	0.32	1.13–2.33	0.082	0.93
Post	5.34	0.14	4.93–5.53	1.53	0.26	1.2–2.07	57.5	< 0.001 *
Paired “t”	68.09			3.98				
P (significance)	< 0.001 *			≤ 0.001 *				

*N*, number; *SD*, standard deviation; St “t” test, Student “t” test, statistically significant P value *p < 0.001

#### Regarding students’ emotional regulation

- The **Cognitive Reappraisal facet (ERQ-R)** Table 4 shows there was no statistically significant difference (*P* > 0.05) between the intervention and control groups regarding the mean values of pre-intervention ERQ-R (13 for both). Conversely, there was a statistically significant difference (*P* < 0.001) between both groups regarding post-intervention values, where the mean value was statistically significantly higher among the intervention than the control group (38.1 and 10.3, respectively). Within each group, there was a statistically significant change in the mean values of ERQ-R, where the post-mean increased among the intervention group (from 13 to 38.1), while the controls showed decreased mean value (from 13 to 10.3) (*P* < 0.001). The decrease in cognitive reappraisal score for the control group may also be attributed to the inherent stressors and demands of their clinical and academic environment.
- For the ***Expressive Suppressive facet (ERQ-S)***, in Table 4, there was no statistically significant difference (*P* > 0.05) between the intervention and control groups regarding the mean values of pre-intervention ERQ-S (22.00 and 21.75, respectively). On the contrary, there was a statistically significant difference (*P* < 0.001) between both groups regarding the post-intervention values, where the mean value was statistically significantly lower among the intervention than the control group (6.55 and 23.6, respectively). Within each group, there was a statistically significant change in the mean values of ERQ-S, where the post-mean value decreased among the intervention group (from 22.00 to 6.55). In contrast, the control group showed an increased mean value (from 21.75 to 23.6) (*P* < 0.001).

**Table 4:** Comparing the studied groups’ cognitive reappraisal and expressive suppression facets pre- and post-intervention

	Intervention group (*N* = 20)			Control group (*N* = 20)			St. “t” test	*P* (significance)
	Mean	SD	Range	Mean	SD	Range		
**Cognitive Reappraisal facet**								
Pre	13.0	2.15	8–18	13.0	1.94	9–16	0.0	1.0
Post	38.1	1.72	34–41	10.3	1.65	7–12	52.06	< 0.001 *
Paired “t”	42.3			6.89				
P (significance)	< 0.001 *			< 0.001 *				
**Expressive Suppression facet**								
Pre	22.0	2.45	18–72	21.75	2.17	18–26	0.41	0.68
Post	6.55	1.23	4–9	23.60	2.06	20–27	31.7	< 0.001 *
Paired “t”	28.4			6.13				
P (significance)	< 0.001 *			< 0.001 *				

*N*, number; *SD*, standard deviation; St “t” test, Student “t” test, statistically significant P value *p < 0.001

### Qualitative data

Three themes were identified and processed through the intervention sessions. The themes were formulated based on the students’ words and reflections on processes. In the transcripts, we refer to Students as “S” and number them from 1 to 20 while referring to the practitioner as “P.”

#### Theme 1: Mindfulness of emotional and mental States

During the first session, nearly all students reported fears of being in a mental health setting or of creating a contact space with patients who suffer from mental issues. Most of the students described a deeply rooted cultural confusion related to the question, “Is it spirits’ or demons’ possessiveness or mental illness?

The practitioner facilitated space to reflect on what each of these interpretations meant to the students in terms of cultural, familial, personal, emotional, and mental states [35]:

S1: *I cannot be in a mental hospital or the same room with a mentally ill person.*

P: *What does it mean to you to be in a mental hospital or even in the same room as a patient exhibiting mental problems?*

S1: *I will lose it. I am already on the edge.*

S19: *My cousin suffered the same features we studied in “the schizophrenia lecture*,*” but we never considered it as a mental disorder! We always consulted religious professionals with their religious tools to help her. Unfortunately*,* that has been going on for years*,* and she has never been helped.*

Some students showed a tendency to apply psychiatric symptomatology on themselves or their families, so it seemed essential to facilitate the process for them to reflect on their fears and what seemed to be cultural, familial, or personal misunderstandings of the core of mental health issues and their manifestations. Thus, around the third and fourth sessions, following the ABC model, the practitioner invited students to recall an experience where they, their families, or someone they knew experienced mental health issues or believed there was some demonic possession going on. Then, the practitioner invited each to recall and describe their beliefs and the mental and emotional states around such events. The experience evolved some deep processes that allowed students to accept and reflect on their new learning experiences related to mental health issues.

S10: *My sister suffered lots of problems that I realize now are psychotic features. Everyone around us*,* including my family*,* believed some demon possessed her. For the first time*,* I was not terrified of contacting her. I feel ready to explain to my family what I have learned and encourage them to take her to a psychiatrist.*

S15: *Before*,* I used to think of mental illness as an infection that I might catch just through talking. I now understand more about the fundamentals of mental illness*,* and my confidence in myself and the professional methods I employ is growing.*

S 5: *During sessions*,* I realized the fear was not mine. It was more an inherent experience I got from culture*,* extended family*,* and the way my mother used to describe my grandfather as possessed by demons / “Jinn.” We never talked about it*,* but there was always a fear inside us that we might become possessed as well.*

S 8: *Two of my family members were described as possessed by demons / “Jinn” and never visited psychiatrists. We were forbidden to communicate with them for fear of becoming possessed. Now*,* I feel able to help myself and my family.*

Facilitating the process for students to reflect on their experiences and articulate what they did not understand or were not allowed to discuss helped them make sense of their processes, develop a space to interact with patients, and allow their experiences to be processed.

#### Theme 2: Emotional regulation

At the beginning of sessions, some students expressed their struggle with recognizing, defining, or regulating emotions such as anger, anxiety, sadness, guilt, and fear. Others described how they could not tolerate silence, especially with patients. The uncertainty of what was going on in patients’ minds, or the impact of their interventions on them, was described as an overwhelming experience. In the second session, mechanisms of regulating emotions – especially cognitive reappraisal and the experience of emotion suppression – were explored:

S12: *I do not know how I feel! No one in my family talks about feelings; it is forbidden.*

*P: What do you mean by forbidden?*

S12: *There is no time to waste on emotions. In our family*,* feelings mean weakness; we used to bury or ignore emotions and get on with whatever happened.*

P: *You used to suppress*,* not feel*,* or express your feelings?*

S12: *When you ask me how I feel*,* I get confused. We are not supposed to let our feelings get in the way. We are here to learn and be professional.*

P: *So basically*,* learning and getting to be professional means not feeling*,* not recognizing*,* and processing emotions. Is that what you mean?*

S12: *Exactly.*

P: *Would you hold that for now and check what happens inside you when I ask you to go deeper and process your feelings?*

S12: *I am trying not to.*

P: *If you try not to suppress them*,* what happens?*

The practitioner invited the students to use silence to check and reflect on their inner processes while observing their emotions and regulating mechanisms and then to consider sharing them with the group. For some, it was difficult as – according to them – they were taught to be strong and never feel or express emotions. A few described how they used to turn all negative feelings into positive ones, telling themselves everything would be ok, and there was nothing to worry about. By contrast, others described how they were invited to be vulnerable and even dramatically emotional.

Regarding the students’ tendency to adopt the “expressive suppression” strategy, the study explored what it meant for each student to suppress their positive or negative emotions. The students presented cultural beliefs about positive emotions that encouraged them to protect their positive feelings from others’ envy or “evil eyes” by keeping things to themselves. About negative feelings, some students stated they had learned to demonstrate negative feelings in their behaviors – such as allowing themselves to be sad and even to cry – but never to express them verbally. At the same time, the majority confirmed that culturally, what they should do is hide (or suppress) negative feelings. Thus, experiencing getting in contact with feelings, recognizing and expressing them, and exploring alternative strategies to regulate them facilitated students’ being more empathetic while creating space for patients to recognize, process, and integrate their feelings.

S2: *I am aware now that while I am in the situation*,* I am gradually developing the ability not to follow my usual negative pattern of interpreting my experience but to shift my state to a more adult*,* matter-of-fact way.*

P: *And what does it mean to you to experience such a shift?*

*S2: I know how that helps me be more available and less emotionally self-centered. I am more aware of the cocreated process I share with others and of my capability to process and shift my internal state and*,* consequently*,* my emotions.*

#### Theme 3: Using self as a therapeutic tool

The subject of using self as a therapeutic tool was raised at the first two sessions as a real struggle for students to process and integrate:

S7: *“Therapeutic relationship? You are the most important tool in the relationship?” I am still trying to understand what that means! I have yet to gain experience to apply all that. I am still figuring out how to use myself as a tool as we were taught. I need more than the theory to enable me to do it.*

S19: I*t is not in our culture*,* the therapeutic relationship*,* or the counseling context. That needs time and experience. How come our first experience will be with someone who may need more ability to engage in any contact?*

By around the fourth session, developing the ability to contact their feelings, be aware of their emotional and mental processes, and adopt an appropriate strategy to regulate emotions enabled students to use their developing skills in facilitating therapeutic processes with patients. Further, practicing the three dimensions of reflection: before, on, and beyond action facilitated students to develop their ability to reflect- in action while in actual situations.

S2: *I want to describe my new experience with tolerating silence. Yesterday*,* when I tried to contact the client assigned to me*,* he again did not respond to my transactions. I was aware the process did not terrify me as it used to do before. I even listened to myself copying the process I experienced here in the group and responded*,* “It seems you don’t want to interact now. If so*,* I understand and will be here for you if it is ok for you”. To my surprise*,* he did not take long and soon started talking to me.*

P: *How was that for you?*

S2: *I felt more professional. I wasn’t afraid; I knew he needed time to process things internally and was willing to facilitate that for the patient.*

## Discussion

The current study’s quantitative and qualitative findings demonstrate the RMER program’s pivotal role in enhancing nursing students’ self-awareness, understanding, and self-regulation. The lack of significant differences in socio-demographic characteristics (age, sex, residence, GPA, marital, employment status, etc.) between the intervention and control groups ensures that the study results are attributable to the intervention rather than pre-existing differences.

The results reveal significant improvements in mindfulness and emotional regulation skills among students in the intervention group compared to the control group. These results align with existing literature that suggests that reflective mindfulness and emotional regulation-based interventions are powerful teaching and learning approaches that enhance mindfulness, emotional regulation, critical thinking, and clinical reasoning [7, 30], empathy [12], personal and professional continuous development, all of which ultimately raises the level of patient care [15, 16]. The program offered students the experience of being in an actual therapeutic relationship and using the self as a therapeutic tool [15, 31]. This was of particular contextual significance as the counseling / psychotherapeutic field was unfamiliar within the culture where the study was conducted.

The decline in mindfulness and cognitive reappraisal scores in the control group underscores the detrimental impact of sustained academic and clinical stress without targeted support. In contrast, the RMERT intervention, integrating mindfulness techniques, TA, and CBT, provided a robust framework to counteract stress. Mindfulness practices promoted present-moment awareness and self-reflection, CBT facilitated cognitive restructuring to challenge negative thought patterns, and TA offered empathic, structured interactions. These combined strategies not only halted the deterioration of adaptive coping skills but also enhanced emotional resilience, highlighting the intervention’s critical value for sustaining well-being among nursing students [10, 14, 20].

In research studies [30, 32], nursing students have described clinical settings as emotionally charged areas where they are expected to manage intense mental and emotional states on their own; in most cases, this exacerbates their feelings when they lack the necessary knowledge, skills, experiences, and supervised self-reflective processes. The current study results agreed with such a claim since the pre-test of both the intervention and control groups displayed similar results, where students showed less mindfulness of their processes, less of a tendency to regulate emotions in terms of adopting the cognitive reappraisal strategy, and more of a tendency to suppress their emotions – whether positive or negative [13, 14]. Furthermore, the post-test results of the control group confirmed the same outcome since results showed a statistically significant decline in students’ responses compared with those of their pre-tests. Students showed less mindfulness by spending time in the clinical setting, a statistically significantly diminished tendency to adopt the cognitive reappraisal strategy, and more of a marked tendency to adopt the suppressive one [33]. In that regard [31, 34], explain how students go through conflicted mental and emotional processes when confronted by experiences that force them to face their deep fears alone without the luxury of preparation.

Though learning is mainly derived from experience [30], confirmed that learning can only be implemented with critical appraisal and reflection. Quantitative and qualitative data were consistent with this since they revealed a notable improvement in intervention group mindfulness. This improvement aligns with previous literature, which suggests that mindfulness training enhances present-moment awareness and reduces emotional exhaustion, especially in high-stress professions like nursing [2]. During sessions, students were facilitated to reflect upon their struggle with recognizing, processing, or verbalizing their mental and emotional states [30, 34]. Initially, they expressed anxieties deeply tied to cultural views, often linking mental health issues to stigma or even supernatural beliefs, reflecting limited mental health literacy [35]. Facilitated reflections allowed students to challenge and reconsider these beliefs, promoting a more clinical and empathetic approach to mental health care [16]. This result aligns with Ličen & Prosen’s, (2023) & Yancey (2018) work on integrating ambiguity and culturally sensitive educational environments that enable students to engage critically with culturally ingrained beliefs. Students reported a developing ability for self-reflection and a decreased fear of mental illness [36, 37].

The study assessed students’ probable tendency to use two of the strategies: cognitive reappraisal (a healthy strategy) and expressive suppression (a maladaptive one) [12, 14]. The contrast between the two strategies enabled assessing, challenging, and promoting students’ mindfulness of their processes [13, 14]. Although expressive suppression can sometimes be helpful in high-stress clinical settings, where emotional control is often needed, for nursing students, relying on suppression without reflective practices may lead to adverse effects. Reflective practices help students process emotions and build empathy [12, 31]. Studies show that suppression alone can limit self-awareness and hinder the development of essential interpersonal skills. For nursing students, balancing suppression with reflection is essential to managing emotions and maintaining resilience in complex clinical environments [12, 20]. Experimental studies [13, 38] have shown that adopting the cognitive reappraisal strategy positively influences the person’s emotional and mental states, decreasing the experience of negative emotions and thus facilitating intrapsychic and interpersonal skills. Conversely, adopting the suppression strategy develops a negative effect, decreasing positive emotional experiences while upholding negative ones.

Students named emotions such as anger, stress, anxiety, depression, and fear as the most overwhelming, especially when they experienced silence, uncertainty, or confusion [32, 39]. In order to address the students’ expressed struggles with silence, uncertainty or confusion, silent technique [40], self-reflection [16, 17], and empathic transactions [25] were utilized to help students reflect upon and become more aware, tolerant, and understanding of their processes as well as to improve their capacity to use these processes and themselves as therapeutic tools. In line with such an approach [37, 41] emphasizes the significance of creating an educational, safe space where students can develop the experience of working with ambiguity and uncertainty and apply it to developing their professional skills.

By these results, the post-test responses and the qualitative data of the intervention group indicated that students developed a greater capacity to engage in mindfulness, demonstrated a greater tendency to feel open towards sharing emotions (both positive or negative), and a lessening of any tendency to suppress their emotions [42, 43]. Research studies suggest that as students develop the ability to process, reflect on their experiences, and develop an empathetic understanding of mental health issues, they can facilitate patients to develop a more integrative sense of self and a probable longed-for growth and development [16, 44, 45].

### Limitations and recommendations

The study has several limitations. The trial’s sample size was small, which may have restricted the potential to apply the findings to other populations of nursing students or healthcare settings. However, considering the nature of the study and intervention, it was more attainable to conduct with a smaller sample size. Replicating the study with more extensive and diverse populations would validate its rigor and generalizability. Due to the small and specific sample from a single institution (Benha University), the results may need to be more generalizable to nursing students from other institutions, regions, or cultural contexts. Replicating the study with more diverse populations could strengthen the findings. The post-hoc power analysis indicated a relatively low power to detect significant effects, which suggests a higher likelihood of a Type II error (failing to detect an effect when one exists). Consequently, caution is warranted when interpreting null findings, and future research should consider larger, more diverse samples to validate and generalize these results.

The small sample size may have contributed to this limitation. Due to the nature of the intervention, it was difficult to blind the researchers and participants. This lack of blinding might introduce bias in the study outcomes, as participants and researchers were aware of which group they were assigned to. The study was conducted in a cultural context where counseling and psychotherapy are not commonly practiced or understood. While this is a significant factor in the study’s relevance, it also limits the applicability of the findings to cultures with different attitudes toward mental health and therapeutic practices. Although the researchers ensured that the control group did not receive any additional interventions during the study, the possibility of contamination (unintended exposure to mindfulness or emotional regulation techniques) cannot be entirely ruled out, especially since both groups attended the same theoretical and clinical training sessions.

To enhance sample size and statistical power in future studies, researchers should consider multi-site collaborations across various nursing programs to widen the recruitment pool and diversify participants. Extending the recruitment period and leveraging digital platforms for enrollment can also attract more participants. Additionally, incorporating longitudinal follow-up designs will allow for repeated measures, which can strengthen power and provide insight into long-term effects. Adopting cluster-randomized or stratified sampling methods and offering incentives for participation may further reduce dropout rates and enhance engagement. These concrete methodological adjustments can mitigate Type II error risks and improve the generalizability of findings.

## Conclusions

This study showed how psychoeducational reflective practices/ programs effectively enhance students’ awareness, understanding, and regulation. The program allowed students to engage in therapeutic and reflective interaction and use themselves as a therapeutic tool. Students were facilitated to reflect on their personal and professional experiences, vital in healthcare promotion, whether for students or patients. Such methods help students evaluate their mental and emotional processes and critically appraise their clinical and professional decisions, which ultimately enhance professional practice.

### Nursing implications

The findings of the RMERT study have significant implications for nursing education, particularly in preparing students for the emotional and psychological demands of mental health nursing. Incorporating Reflective Mindfulness and Emotional Regulation Training (RMERT) into nursing curricula can help students develop essential skills such as mindfulness, self-awareness, and emotional regulation. These skills are crucial for managing the inherent stress and anxiety associated with clinical practice, particularly in mental health settings. Using Schön’s reflective dimensions (before, during, after, and beyond action) in the RMERT program highlights the importance of fostering critical thinking and self-improvement, which can be systematically integrated into theoretical and clinical components of nursing education.

One of the significant challenges nursing students face is understanding and using themselves as therapeutic tools [46, 47]. RMERT addresses this by enabling students to process their emotional and mental states, which improves their ability to establish therapeutic relationships. Furthermore, the program emphasizes empathy training, allowing students to support patients better while maintaining their emotional well-being. Addressing cultural stigmas and norms regarding emotional expression is also critical. Many students in the study grappled with cultural misconceptions about mental illness and emotional regulation. By facilitating open discussions about these stigmas, nursing education can equip students to navigate diverse patient perspectives with sensitivity and confidence.

Additionally, RMERT helps reduce stress and anxiety by teaching students to manage emotions effectively, enhancing academic performance and clinical competence. Introducing mindfulness exercises, role-playing scenarios, and group counseling sessions into the curriculum can create a supportive learning environment where students feel safe to explore their vulnerabilities. These approaches improve students’ ability to tolerate ambiguity and silence during therapeutic interactions, fostering professionalism in their patient care.

Evaluating students’ progress through tools like the Mindful Attention Awareness Scale (MAAS) and the Emotion Regulation Questionnaire (ERQ) can ensure the program’s efficacy. By integrating RMERT principles into nursing education, educators can prepare students to provide empathetic, high-quality care while promoting personal and professional growth. This approach is essential for addressing modern healthcare’s emotional and psychological challenges and cultivating resilient, emotionally intelligent practitioners.

## Data Availability

Data will be available upon reasonable request from the corresponding author.
